# Healthy Life Expectancies by the Effects of Hypertension and Diabetes for the Middle Aged and Over in Taiwan

**DOI:** 10.3390/ijerph17124390

**Published:** 2020-06-18

**Authors:** Chia-Chun Liang, Wei-Chung Hsu, Yao-Te Tsai, Shao-Jen Weng, Ho-Pang Yang, Shih-Chia Liu

**Affiliations:** 1 Department of Radiation Oncology, Chung-Kang Branch, Cheng-Ching General Hospital, 40764 Taichung, Taiwan; wdataul2003@gmail.com (C.-C.L.); b751106@yahoo.com.tw (W.-C.H.); 2 Department of Occupational Therapy, Asia University, 41354 Taichung, Taiwan; 3 Department of International Business, Feng Chia University, 40723 Taichung, Taiwan; yaottsai@fcu.edu.tw; 4 Department of Industrial Engineering and Enterprise Information, Tunghai University, 40704 Taichung, Taiwan; sjweng@thu.edu.tw; 5 Healthcare Systems Consortium, Tunghai University, 40704 Taichun, Taiwan; 6 Department of Cardiology, Show Chwan Memorial Hospital, 50008 Changhua, Taiwan; yanghopang@yahoo.com.tw

**Keywords:** hypertension, diabetes mellitus, disability, healthy life expectancy

## Abstract

(1) Introduction: This study aims to investigate the disparity in the healthy life expectancy of the elderly with hypertension and diabetes mellitus. (2) Materials and Methods: This study used survey data collected in five waves (1996, 1999, 2003, 2007, and 2011) of the “Taiwan Longitudinal Study on Aging” (TLSA) to estimate the life expectancy and healthy life expectancy of different age groups. The activities of daily living, the health condition of hypertension and diabetes and the survival statuses of these cases were analyzed by the IMaCh (Interpolated Markov Chain) and logistic regression model. (3) Results: As regards the elderly between age 50 and 60 with hypertension and diabetes, women with hypertension only exhibited the longest life expectancy, and the healthy life expectancy and the percentage of remaining life with no functional incapacity were 33.74 years and 87.11%, respectively. In contrast, men with diabetes only showed the shortest life expectancy, and the healthy life expectancy and the percentage of remaining life with no functional incapacity were 22.51 years and 93.16%, respectively. We also found that people with diabetes showed a lower percentage of remaining life with no functional incapacity. (4) Conclusions: We suggest that policymakers should pay special attention to publicizing the importance of health control behavior in order to decrease the risk of suffering diseases and to improve the elderly’s quality of life.

## 1. Introduction

Along with the progress and improvement of the social economy and health care, the life expectancy in Taiwan is increasing annually; in particular, the increase in lifespan for the elderly aged above 65 is more obvious than for other age groups. In May 2020, the elderly above 65 populations in Taiwan is 15.62% of the total population (the senior society), and the ratio would exceed 20% to achieve the aged society in 2025 [1]. It is worth noting that it will take about 25 years to move from a senior society from aging society in Taiwan, which is about the same as in Japan but faster than the 115 years necessary in France, the 72 years necessary in the USA and the 47 years necessary in the UK. In this case, the care and nursing demands for the elderly population are becoming the primary issues of concern for the government, as the increase of the elderly’s healthy life years and the maintenance of quality of life are the critical issues encountered by an aging society.

On the other hand, chronic diseases are the major factor in middle-aged and elderly death, including malignant tumors, cardiac disease, cerebrovascular disease, diabetes and hypertension. The middle-aged and elderly, with their reducing metabolic capability, can easily suffer from chronic diseases and their complications and face increased risks of death. Apparently, the health burden caused by chronic diseases will be increasingly present as the society ages. This will affect individual physical and mental conditions and might increase family expenses resulting from medical treatment.

The development of a person’s health conditions starts with suffering from a disease and then leads to death. In other words, suffering from a disease is the starting point of health degradation. The elderly’s health conditions are a concern of the senior society, where chronic diseases are the major factors in the function of activity and self-rated health [2]. A large amount of relevant research has also discussed the effects of middle-aged and elderly chronic diseases, physical functions and lifestyles on health change [3,4,5,6,7,8,9]. Among various diseases, diabetes is a common chronic disease in the elderly population. Cerebrovascular disease, cardiovascular disease and kidney disease, which are among the 10 leading causes of death in Taiwan, are closely related to poor diabetes control. It has been shown that diabetes has huge effects on citizens.

According to the statistical annual report of medical care by the National Health Insurance, the health insurance claims for diabetes in Taiwan amounted to about 1.9 million person-times in 2015—about 200 times the death toll in the year—where the visit rate per 0.1 million population appeared to be the highest for the elderly aged above 65, at 28,295 people, which was about 3.5 times the general average. On the other hand, the average health insurance medical fee point per person for diabetes was 10,438 in 2015 (around NTD 9,500 or USD 317), which was not the highest medical expenditure but was significant when including the medical fees for related complications [10]. The Taiwanese Association of Diabetes Educators survey in 2005 showed that 67% of diabetic patients were suffering from hypertension and that patients with hypertension could easily suffer from diabetes [11]. Their research also pointed out the continuous relationship between blood pressure and the occurrence of stroke or other cardiovascular diseases for patients with hypertension, and the risk factors should be strictly controlled—i.e., blood pressure—to avoid stroke or other cardiovascular diseases [12]. Hypertension is the most important factor of the multiple risk factors of stroke and can be cured and controlled to effectively prevent a first-time or repeated stroke. Diabetes is currently regarded as a metabolic disease, which often accompanies symptoms of hypertension, obesity, high-density, lower cholesterol and high triglyceride [13]. Thus, diabetes should be noted in the beginning or early stage. Nevertheless, the early symptoms of hypertension and diabetes are not obvious; some patients do not realize they are suffering from diabetes or hypertension after an acute cardiovascular disease or other complications [14,15]. The indicators of abdominal obesity, hypertension, hypertriglyceridemia and hyperglycemia are positively tested in Taiwan for the prevention and control of metabolic syndrome. For patients with cardiovascular disease, increasing physical activity and reducing bad habits—e.g., smoking—could effectively improve the physical conditions and further reduce death risks [16,17,18]. Furthermore, an increase in physical activity could effectively promote patients’ quality of life and self-perceived health as well as encourage patients to seek medical advice or employ prevention measures to reduce the harm caused by chronic diseases [17,19]. Relevant research has also indicated that the elderly with diabetes and hypertension would experience a decreased quality of life and social function and further result in increasing mortality [20]. As a result, it is an important challenge for public health and governmental departments to make related policies to maintain the health of the elderly.

Population aging and lengthened lifespans do not necessarily show the promotion of quality of life. Nevertheless, disability is not an inevitable part of life in the aging process of the population [21,22]. As a result of the aging process, expenses for relevant medical care and long-term care would be increased in a population with limited physical functions or with increasing age. Since 2013, the life expectancy without relying on others for physical health has been used for the measurement of national physical health to reflect the overall national health conditions and has become a statistical indicator of gross national happiness in Taiwan [23]. The healthy life expectancy is broadly utilized to measure the health and trends of the population and is not restricted to the measurement of disabilities but can be calculated with different health needs. Lin and Liu estimated the healthy life expectancy of the elderly suffering from chronic diseases in Taiwan [24]; Lin and Liu also discussed healthy life expectancy focusing on the successful and active aging of the elderly in Taiwan [25].

If extending life expectancy is a global goal to enhance social welfare, reducing disability years and increasing healthy life expectancy is a primary issue and challenge encountered in an aging society. Moreover, it should be determined whether the quality of life of the elderly would be promoted by economic development and medical advances in Taiwan. Taking healthy life expectancy without disabilities as the indicator of health conditions in this study, the difference between the life expectancy and healthy life expectancy of the elderly suffering from hypertension and diabetes in Taiwan is compared to discuss the effect of important chronic diseases on healthy life expectancy.

## 2. Materials and Methods

### 2.1. Data

The “Taiwan Longitudinal Study in Aging” (TLSA) conducted by the Health Promotion Administration, Ministry of Health and Welfare was used as the data source. Originally, the registered population aged above 60 by 1988 in Taiwan (not including mountain townships) was sampled with the stratification of multi-level sampling for TLSA. After completing the baseline survey in 1989, a follow-up visit was completed every 3–4 years. In order to establish the complete database for those above the age of 50, supplementary samples with lower age groups were sampled, as in 1989, in 1996 and 2003; the follow-up visit rate was above 80%. The major surveyed issues contained health conditions, social support and the working and economic statuses of cases.

Regarding the selection of research objects, the cases in 1996 are selected as the major research objects (total 5131 cases aged above 50) for this study. The life expectancy and the healthy life expectancy are estimated according to the situations of suffering from hypertension and diabetes, the activities of daily living (ADLs) and survival conditions in the survey in 1996, 1999, 2003, 2007 and 2011.

In terms of overall health evaluation, disability-free life expectancy is defined as healthy life expectancy; the activities of daily living (ADL) scale is used to measure the health state. The scale covers the following activities:

- Taking showers;
- Putting on clothes or taking off clothes;
- Having meals;
- Getting up, standing or sitting on chairs;
- Walking indoors;
- Going to the toilet.

A case where any activity is assessed as slightly difficult, very difficult or completely impossible to do is defined as disability (unhealthy state). Disease is defined by self-reporting for a case of suffering from diabetes or hypertension.

### 2.2. Research Method 

The discrete-time Markov chain model is used as the analysis model to calculate healthy life expectancy, and the maximum likelihood method is utilized to estimate the transfer probability of health status and calculate the healthy life expectancy [26]. Based on the theory, Lièvre et al. developed the IMaCh software to calculate healthy life expectancy [27]. The software could fully apply all information of a case, including the date of birth, date of death and survey date, as well as deal with losing contact with cases, including due to the dynamic change of health state and the loss of survey data. This method is also suitable for tracking situations with different survey intervals.

With IMaCh, the health state, status of suffering from a disease and survival conditions of the middle-aged and elderly in Taiwan were used to estimate the life expectancy and healthy life expectancy. For the estimation of life expectancy and healthy life expectancy with IMaCh, the discrete-time Markov chain was used as the analysis structure; in addition, the logistic regression model was utilized and the required covariance was added for the analyses, including the year and month of birth, year and month of death, visit completion time in various surveys, health state (i.e., disability situation) in various surveys and status of suffering from a disease in the various case surveys. The statistical model and theoretical structure were determined by the IMaCh software, and the analysis programming proceeded according to the website of Computing Health Expectancies using IMaCh (http://euroreves.ined.fr/imach/).

**Figure 1:**
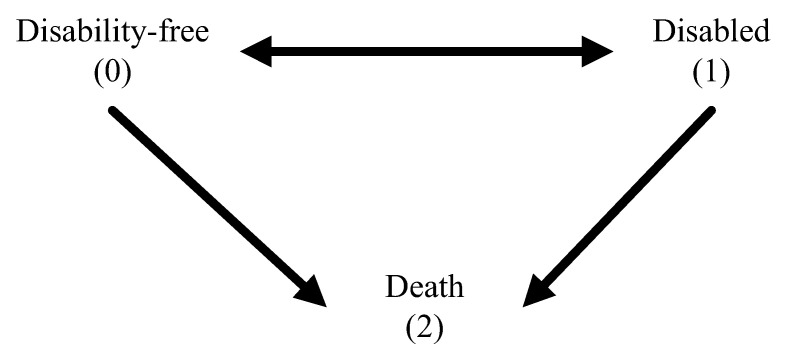
States and possible transitions across states.

According to the effects of the elderly in Taiwan suffering from chronic diseases and their related factors on healthy life expectancy discussed in this study, the polychotomous logistic regression model used in IMaCh is shown as below:

(1) $$\begin{array}{cc} ln\frac{P_{jk}\left(x,\;h\right)}{P_{jj}\left(x,\;h\right)}= & \alpha_{jk}\left(h\right)+\beta_{1jk}\left(h\right)*x+\beta_{2jk}\left(h\right)X_{1}\left(h\right)\\ & +\beta_{3jk}\left(h\right)X_{2}\left(h\right)+\beta_{3jk}\left(h\right)X_{4}\left(h\right),\;j\neq k \end{array}$$

where *x* is the age of the case, $X_{1}$, $X_{2}$, and $X_{3}$ are covariances representing gender (male, female), hypertension (suffer from the disease or not) and diabetes (suffer from the disease or not), respectively, and $P_{jk}\left(x,\;h\right)$ reveals the transfer probability of state *j* of the case aged *x* being the state *k* after the time *h*. The health state change of cases in the tracking period is shown in Figure 1, including “healthy” (without ADL disability) and “unhealthy” (ADL disability), which are mutually reversible, and the irreversible “death”; the state “0” represents a health state without disability, “1” represents the unhealthy state with physical disability and “2” shows death. As a consequence, the transfer probability of various states could be acquired according to the model estimate; this is the basis of estimating the individual health state and healthy life expectancy with IMaCh. The relevant theoretical basis can be found in the research work by Lièvre et al. [27].

In summary, the situation of the middle-aged and elderly in Taiwan suffering from hypertension and diabetes is mainly discussed in this study. The life expectancy and healthy life expectancy are calculated according to health conditions, the situation of suffering from a disease and survival conditions in the surveys from 1996 to 2011; basic demographic variables of gender and age are also included to adjust the effect of the conditions of suffering from a disease on health.

## 3. Results

Table 1 shows the sample characteristics surveyed in 1996. Most cases were male (52.96%) and aged below the age of 60 (40%). Regarding the situation of suffering from major chronic diseases, 24.68% and 10.59% were represented by hypertension and diabetes, respectively. About 90% of respondents showed normal ADL conditions.

Table 2 displays the surveyed ADL disability ratio in 1996–2011. According to gender, hypertension and diabetes, the surveys reveal a larger percentage of disability for females than males and a higher percentage of disability for patients suffering from chronic diseases. On the other hand, the research results also show that, regardless of gender, those suffering from hypertension and diabetes appear to experience the most serious disability situation in various survey years. As an example, in 2011, cases suffering from hypertension and diabetes showed 25.49% and 34.84% of ADL disability for males and females, respectively.

According to the situation of suffering from the chronic diseases of diabetes and hypertension and gender, Table 3 shows the percentages of life expectancy and healthy life expectancy of the middle-aged and elderly aged 50, 60, 65, 75 and 85 surveyed in 1996–2011. Overall, females present a higher life expectancy than males, but the trend of healthy life expectancy percentage appears to be the opposite. As an example, for cases aged 50, regardless of whether the respondents were suffering from diabetes or hypertension, females showed a higher life expectancy of 28.01–33.74 years than males, at 22.51–29.58 years, while the female healthy life expectancy percentage, 84.19–89.28%, was lower than the male healthy life expectancy, 91.08–94.21%.

With regard to the situation of suffering from a disease, cases suffering from diabetes, regardless of the presence of hypertension, presented a higher life expectancy and healthy life expectancy percentage than those not suffering from the disease. On the other hand, cases suffering from hypertension showed higher life expectancy but not a healthy life expectancy than those not suffering from the disease. For instance, a 50-year-old male case suffering from diabetes but not hypertension had a life expectancy, healthy life expectancy and healthy life expectancy of 22.51 years, 20.97 years and 93.16%, respectively, while cases with hypertension showed values of 24.09 years, 21.94 years and 91.08%, respectively. On average, the life expectancy between cases with/without suffering from hypertension showed a 1 year difference, and there was an about 2% difference in healthy life expectancy. This phenomenon is also reflected by female cases; that is, those suffering from hypertension might lengthen their life expectancy with proper care, but life-extension does not enhance the healthy life expectancy, as an extended lifespan with hypertension could be regarded as an unhealthy life expectancy. Furthermore, research results also show that cases with/without suffering from hypertension show a larger difference in healthy life expectancy with increasing age.

## 4. Discussion

The proportion of the elderly aged above 65 in Taiwan has been increasing since 1993, reaching 13.20% in 2016 and 14.05% by March 2018, leading Taiwan to formally become a senior society. In comparison with advanced countries in Europe and America, the aging of the population was rather fast. Relevant departments should therefore positively face the impact and effect of the aging society, including long-term care and economic and social welfare development, to cope with the major issue of aging.

When considering the effect of population aging on the entire society—particularly, the faster aging of the population in Taiwan than in other countries—it must also be acknowledged that hypertension and other cardiovascular diseases are the common chronic diseases of the middle-aged and elderly. Diabetes, as with a metabolic disease, is related to hypertension, cardiovascular disease, hyperlipidemia and kidney disease, which are tracked and studied in the elderly in Taiwan. With the analysis of healthy life expectancy, and aiming at the effect of suffering from the major chronic diseases of hypertension and diabetes on health, the life expectancy and healthy life expectancy of the elderly in different generations are estimated, and the measurement of the quality of life of the elderly in old age (the healthy life expectancy percentage) is further provided.

In comparison with males, females experience more restrictions and fewer resources in life, meaning that negative effects could more easily appear in the old age; the encountered problems, therefore, would be highlighted in old age. The longer life expectation also means that females show a worse health and quality of life than males. The analysis results of this study could provide evidence for this. Current medical care resources and economic security system design should therefore take gender into account. Economic security, living models, home care and social support to promote female elderly quality of life in old age and the relevant issues should be focused on by citizens.

On the other hand, the risk of suffering from hypertension and diabetes increases with increasing age. It is shown in this study that diabetic patients show a lower life expectancy and healthy life expectancy percentage, revealing the worse quality of life of diabetic patients in Taiwan in old age. This is related to diabetic patients more easily developing other chronic complications. When the situation is not properly controlled, it can easily induce cardiac disease and stroke, resulting in complications of the eyes, kidney and lower extremity blood vessels, and can even cause blindness, kidney dialysis and amputation. Diabetic patients, therefore, have to regularly receive various examinations. The early discovery of lesions and early treatment could avoid the threat to life and effects on quality of life. Furthermore, being diagnosed as suffering from hypertension might be a protective factor for patients, as they would listen to physicians’ suggestions to improve their health behaviors and further reduce death risks [14,15]. The research results reveal that, regardless of suffering from diabetes or not, patients with hypertension show a higher life expectancy than those without hypertension (but with a smaller difference), but the healthy life expectancy percentage is not higher. In other words, the increased life expectancy is unhealthy life expectancy, revealing the effect of hypertension on the health quality of citizens in old age. In this case, when extending lifespans, the maintenance of quality of life should be considered, meaning that life expectancy in Taiwan is enhanced while the elderly quality of life in old age is not promoted with social economy and medical improvement. This is undoubtedly a warning for Taiwan.

Health is a basic human right. Along with time advance, the citizens stress more on personal health and security that lengthening and improving national healthy life expectancy and maintaining the quality of life are the expected goals of health policies. Moreover, chronic diseases are the main cause of elderly health and major diseases for the public, particularly cardiovascular disease, diabetes, and hypertension-related diseases. Cardiovascular disease does not appear obvious symptoms in the early stage that it is easily ignored. However, in consideration of the disease burden, it is a major disease that could not be ignored. In terms of diabetes, improper control of diabetes would result in complications of cardiac disease, stroke, kidney disease, retinopathy, and amputation to seriously affect people’s health and huge disease burden. Although the government continuously promote diabetes joint care network to promote diabetes care quality, including glycated hemoglobin, blood lipids, retina, kidney disease examinations of diabetic patients at least once a year, and diabetes quality payment service programs, there is space for progress.

With the legal regulations and health promotion of the Ministry of Health and Welfare in past years, risk factors in cardiovascular disease behavior control have been improved. For the elderly to have daily independence and autonomous ability, prevention from diseases is considered to be the most economical method, in addition to the promotion of prevention service rather than treatment after the onset of disease. From the viewpoint of economics, improving physical conditions through effective behavior control (e.g., improving eating habits, reducing bad health behaviors and cultivating the habit of regular exercise) could directly reflect on patients’ health indicators (e.g., blood pressure, cholesterol, blood sugar and obesity). From the perspective of a governor, improving physical conditions through effective behavior control could be regarded as an economic behavior in that the importance of health behavior control should be reinforced, such as by increasing physical activity and social activity, to effectively promote quality of life and self-perceived health and assist in the promotion of social support. In addition, to reduce risks of cardiovascular disease and even other related diseases, the economic burden caused by related diseases could be further reduced to maintain elderly healthy life in old age. Integrating effective economic behavior into policies, therefore, is important for the decision-making authorities.

## 5. Conclusions

In conclusion, the results of analyzing TLSA indicated that the life expectancy of females would be higher than males in the middle-aged and elderly group. By contrast, males would have a higher percentage of remaining life with no functional incapacity than females. In other words, females have worse health conditions than males in the elderly group. Regarding the disease status under the same condition as age and gender, the elderly with diabetes would have a shorter healthy life expectancy than the elderly with hypertension. However, the elderly with hypertension have a lower percentage of remaining life with no functional incapacity than the elderly with diabetes. Our findings could contribute to encourage self-promotion for the elderly and provide insights for healthcare decision-makers.

## Figures and Tables

**Table 1:** Characteristics and status of respondents at interview in 1996 (*n* = 5131).

Variable	Value	*n*	% *
Age	50–54	602	16.35
	55–59	817	22.19
	60–64	675	18.30
	65–69	921	16.86
	70–74	1046	13.02
	75+	1070	13.28
Gender	Female	2371	47.04
	Male	2760	52.96
Hypertension	No	3705	75.32
	Yes	1344	24.68
Diabetes	No	4540	89.41
	Yes	559	10.59
ADL	Healthy	4455	89.81
	Unhealthy	671	10.13

* All percentages were weighted. ADL = activities of daily living.

**Table 2:** The percentage (%) of respondents middle-aged and over who were disabled from 1996 to 2011.

Gender	Hypertension	Diabetes	Year				
			1996	1999	2003	2007	2011
**Male**	**No**	No	5.71	4.80	4.34	5.80	15.28
		Yes	11.48	6.71	12.10	10.25	12.96
	Yes	No	11.57	7.76	8.89	11.07	12.24
		Yes	26.51	12.97	17.06	21.23	25.49
Female	No	No	9.32	4.88	6.76	10.28	12.82
		Yes	17.95	12.16	19.63	32.87	27.41
	Yes	No	17.27	10.18	13.66	17.30	21.01
		Yes	24.31	21.77	23.54	24.09	34.84

Note: All percentages were weighted.

**Table 3:** Association outcomes of respondents who were middle-aged and over in Taiwan.

Age	Gender	Male				Female			
	Hypertension	No		Yes		No		Yes	
	Diabetes	No	Yes	No	Yes	No	Yes	No	Yes
50	LE	28.32	22.51	29.58	24.09	33.20	28.01	33.74	28.97
	HLE	26.68	20.97	27.39	21.94	29.64	24.37	29.39	24.39
	%	94.21	93.16	92.60	91.08	89.28	87.00	87.11	84.19
60	LE	20.26	15.43	21.21	16.59	24.36	19.87	24.71	20.55
	HLE	18.55	13.78	18.97	14.36	20.75	16.13	20.33	15.91
	%	91.56	89.31	89.44	86.56	85.18	81.18	87.27	77.42
65	LE	16.60	12.36	17.37	13.30	20.22	16.19	20.45	16.71
	HLE	14.86	10.65	15.11	11.02	16.58	12.40	16.09	12.07
	%	89.52	86.17	86.99	82.86	82.00	76.59	78.68	72.23
75	LE	10.29	7.79	10.71	7.89	12.81	9.94	13.55	10.20
	HLE	8.51	5.98	8.47	5.58	9.22	6.15	9.30	5.72
	%	82.70	76.77	79.08	70.72	71.98	61.87	68.63	56.08
85	LE	5.68	4.02	5.84	4.26	7.78	5.59	7.16	5.73
	HLE	3.94	2.17	3.75	2.05	3.86	2.07	3.38	1.75
	%	69.37	53.98	64.21	48.12	49.61	37.03	47.21	30.54

Note: LE = life expectancy; HLE = healthy life expectancy; % = percentage of remaining life with no functional incapacity.
